# 

**Published:** 1881-04

**Authors:** 


					﻿Whole. Number,
CCXXXVII.
APRIL, 1881.
Number 9,
Volume XX.
THE
BUFFALO
Medical and Surgical Journal.
EDITORS:
TKOS. LOTHROP, M.	A. R. DAVIDSON, M.
P. W. VAN PEVMA, M. D„	EUCIENJ HOWE, M. D.,
HERMAN MANTER, M. ».
BUFFALO :
Published by the Medical Journal Association.
Read the Notice of this Journal among the Advertisements.
Entered at the Post-Office at Buffalo, N. Y., as Second-Class Mail Matter.
				

